# Cancer/testis antigen expression and co-expression patterns in Gastroesophageal Adenocarcinoma

**DOI:** 10.21203/rs.3.rs-4499622/v1

**Published:** 2024-06-20

**Authors:** Sukumar Kalvapudi, Akhil Goud Pachimatla, R.J. Seager, Jeffrey Conroy, Sarabjot Pabla, Sarbajit Mukherjee

**Affiliations:** Roswell Park Comprehensive Cancer Center; Roswell Park Comprehensive Cancer Center; LabCorp (United States); LabCorp (United States); LabCorp (United States); Roswell Park Comprehensive Cancer Center

**Keywords:** Cancer/testis antigen, Gastroesophageal adenocarcinoma, co-expression of CTAs, Immunotherapy

## Abstract

Gastroesophageal adenocarcinoma (GEAC) poses a significant challenge due to its poor prognosis and limited treatment options. Recently, Cancer/testis antigens (CTAs) have emerged as potential therapy targets due to their high expression in tumor cells and their immunogenic nature. We aimed to explore the expression and co-expression of CTAs in GEAC. We analyzed 63 GEAC patients initially and validated our findings in 329 patients from The Cancer Genome Atlas (TCGA) database. CTA expression was measured after RNA sequencing, while clinical information, including survival outcomes and treatment details, was collected from an institutional database. Co-expression patterns among CTAs were determined using Pearson correlation analysis. The majority of the study cohort were male (87%), Caucasian (94%), and had stage IV disease (64%). CTAs were highly prevalent, ranging from 58–19%. The MAGE gene family showed the highest expression, consistent across both cohorts. The correlation matrix revealed a distinct cluster of significantly co-expressed genes, including MAGEA3, NY-ESO-1, and others (0.27 ≤ r ≤ 0.73). Survival analysis revealed that individual CTAs were associated with poorer survival outcomes in patients not receiving immunotherapy while showing potential for improved survival in those undergoing immunotherapy, although these findings lacked robust reliability. Our study provides a comprehensive characterization of CTA expression and co-expression in GEAC. The strong correlation among CTAs like MAGE, NY-ESO-1, and GAGE suggests a potential for therapies targeting multiple CTAs simultaneously. Further research, including prospective trials, is warranted to assess the prognostic value of CTAs and their suitability as therapeutic targets.

## 1. Introduction

Gastroesophageal adenocarcinoma (GEAC) is a heterogeneous disease and encompasses cancer affecting the distal esophagus, the gastroesophageal junction, and the stomach [1]. GEAC is often diagnosed in the locally advanced stage or after metastasis [2]. Only 5.5% of patients diagnosed with metastatic GEA are alive after five years [3]. Furthermore, individuals with locally advanced disease face a significant risk of relapse after curative surgery, leading to an unfavorable prognosis [4]. In 2023, the American Cancer Society anticipates approximately 16,120 deaths due to esophageal cancer and 11,130 deaths attributable to gastric cancer in the United States [5, 6]. Therefore, there is a critical need for research into treatment options to address the challenges posed by GEAC.

Previously, systemic chemotherapy was the standard of care for GEAC, but recent advances in the understanding of the role of the immune system in cancer biology revolutionized care. Combining platinum-based chemotherapy with immune checkpoint inhibitors (ICIs) targeting PD-L1 has become the standard of care, resulting in improved survival [7]. Additionally, research into the molecular mechanisms driving GEAC has led to the development and FDA approval of the use of targeted agents such as trastuzumab in Her2-positive GEAC [7]. However, despite the success of ICIs and targeted therapies, not all patients derive benefit, and others develop resistance to therapy over time [8]. As a result, research is underway to identify markers that can predict positive treatment responses and to develop drugs targeting these novel therapeutic markers. One group of potential targets are cancer/testis antigens (CTAs).

CTAs are a group of antigens consisting of more than 700 individual proteins and 200 associated genes [9, 10]. CTAs exhibit high expression levels during embryonic development, promoting processes such as cell proliferation, migration, and survival [9]. Following birth, most CTAs are restricted to expression only in germ cells and trophoblast tissue. Most importantly, CTAs are also overexpressed in numerous types of tumors, including gastric and esophageal cancers [11]. Examples of CTAs shown to be expressed in gastroesophageal cancers include those from the MAGE family, GAGE family, NY-ESO-1, and MLANA. Recent findings indicate that the elevated expression of oncogenic CTAs amplifies the tumorigenicity and motility of cancer cells, promotes metastasis, and contributes to drug resistance [9, 12, 13]. Detection of CTA overexpression is also associated with reduced survival in many different cancer types [14].

The testis and placenta are sites of CTA expression and are designated as immune-privileged areas. This designation means that the introduction of antigens does not cause a local immune-mediated inflammatory response. Due to the limited interaction between the immune system and CTA proteins in these sites, when expressed elsewhere in the body, these proteins are perceived as “non-self”, leading to the intrinsic immunogenicity of CTAs [15, 16]. As CTAs are inherently immunogenic tumor-specific proteins with a restricted pattern of expression in other tissue types, they are an attractive target for cancer immunotherapy [17]. CTA-based immunotherapy, whether through oncolytic virus therapy, cancer vaccines, or adoptive cell transfer, is a promising area of exploration for further research.

Most ongoing trials for CTA-based immunotherapy in gastric or esophageal cancer are either in preclinical stages or early Phase II. Oncolytic viruses modified to carry the MAGE-A3 gene have been confirmed to be safe for use and are currently being trialed in esophageal and gastric cancer patients (NCT02285816) [18]. Several peptide vaccines targeting NY-ESO-1 in esophageal cancer have undergone phase I clinical trials, revealing evidence of their safety and demonstrating some favorable clinical outcomes including a reduction in tumor size (NCT00106158, NCT01003808). T-cell receptor therapies (TCR-T) designed to target MAGE-A1 and MAGE-A4 are currently undergoing phase II trials (NCT05430555, NCT04752358). Additional details of ongoing and completed clinical trials targeting CTAs can be found in an article by Ai et al [11].

A major challenge in designing trials targeting CTAs in GEAC is the lack of comprehensive data illustrating their expression patterns in these cases. Existing data is limited to demonstrating the expression pattern of one or two CTA antigens in either gastric or esophageal cancer. An additional challenge is that it has been previously demonstrated that expression patterns for individual CTAs vary with the stage and clinical course of the disease. Some patients exhibit stable expression over time whereas others show antigen loss [19]. For example, Fujiwara et al. demonstrated decreased expression of NY-ESO-1 with a reduction in the stage of gastric cancer [20]. Therefore, designing specific therapies to target one or two CTAs may prove ineffective. A potential solution to address these challenges is to develop targeted therapies that simultaneously affect multiple CTAs. However, before proceeding, it is essential to determine the expression levels of CTAs in GEAC and the extent to which they are co-expressed.

To identify GEAC patients who may benefit from CTA-targeted therapy, we sought to assess the CTA expression and co-expression landscape using a primary cohort of 63 patients from Roswell Park Comprehensive Cancer Institute and a validation cohort of 329 patients from The Cancer Genome Atlas (TCGA) database.

## 2. Materials and Methods

### 2.1. Patients and Clinical Data

The primary study cohort was composed of samples from 63 GEAC patients. We elected to analyze esophageal, gastroesophageal junction, and gastric adenocarcinoma together based on their similar molecular features and clinical outcomes in prior research studies [21, 22]. Gastroesophageal junction cancer was defined using the Siewert classification [23]. Specimens were collected under an institutional banking policy (institutional review board protocol I115707) with informed patient consent. This study was approved by Roswell Park Cancer Institute internal review board review (institutional review board protocol BDR 117219) according to institutional policy for nonhuman subject research.

### 2.2. Quality Assessment of Clinical FFPE Tissue Specimens

Sample FFPE blocks were cut into sections of 5μ^ι in thickness on positively charged slides. A single section from each block was H&E stained and assessed for tumor representation adequacy, signs of necrosis, tissue preservation quality, and any fixation or handling issues by a board-certified anatomical pathologist. Specimens with less than 5% tumor tissue content or greater than 50% necrosis were removed from analysis. Tissues from 3–5 unstained sections were required to meet assay RNA requirements (10 ng), with or without tumor microdissection.

### 2.3. Nucleic Acid Isolation and Gene Expression

RNA was extracted from each sample and the expression of the seventeen CTA genes was assessed by RNA-seq, as previously described [24, 25]. RNA (RiboGreen staining) was quantified by a Qubit fluorometer (Thermo Fisher Scientific, Waltham, MA, 02188, USA). On samples meeting validated quality control (QC) standards, gene expression was measured using RNA sequencing of 395 transcripts. RNA libraries were sequenced to appropriate depth on the Ion Torrent S5XL sequencer (Thermo Fisher Scientific, Waltham, MA, 02188, USA).

### 2.4. Data Analyses

Absolute RNA-seq reads for each transcript were calculated using the Torrent Suite plugin immuneResponseRNA (Thermo Fisher Scientific, Waltham, MA, 02188, USA) [24, 25]. The absolute read count from the non-transcript control (NTC) was subtracted from the absolute read counts for the same transcript in all other samples of the same batch to remove library preparation background reads. Normalized reads per million (nRPM) were generated from each background-subtracted read count by comparing each housekeeping (HK) gene background-subtracted read against a previously determined HK RPM profile. Gene expression ranks were calculated for each CTA gene by converting nRPM expression values to a percentile rank between 0 and 100, as compared with a reference population of 735 solid tumors spanning 35 histologies [24, 25]. Pearson correlations were calculated to assess the co-expression of each of the seventeen CTA genes. Unsupervised hierarchical clustering with Pearson’s correlation (R) as a measure of distance was initially used to visualize the gene expression landscape of fifteen checkpoint genes across the patient cohort as a heatmap. Survival analysis for the primary cohort was performed using the Kaplan-Meier method. High and low CTA expression groups were identified using the median value of each gene expression rank as a threshold. Survival analyses were conducted for both the overall cohort and for patients stratified by whether they received treatment with ICIs or not.

### 2.5. External Validation

These analyses were repeated in a 329-patient validation cohort obtained from The Cancer Genome Atlas (TCGA). Only 11 out of the 17 CTA genes assessed in the primary cohort had available data and were investigated.

## 3. Results

The cohort of sixty-three GEAC patients was primarily composed of males (87.3%) and patients aged between 60 and 69 years (38.1%). Most patients were white (93.7%) and had stage 4 disease (63.5%) or stage 3 disease (12.7%). Forty-one biopsies (65.1%) were from a primary tumor and twenty-one patients (33.3%) had a metastatic site biopsied. The esophagus was the most common tumor location (58.7%) followed by the stomach (23.8%), and gastroesophageal junction (17.5%). Most of the patients had an overweight BMI (71.4%) and a positive PD-L1 IHC status (61.9%). Eighteen patients in the cohort were treated with an immune checkpoint inhibitor (286%). Characteristics of the clinical cohort are summarized in Table 1.

Additionally, we identified 329 GEAC patients from the TCGA database to validate the findings in the clinical cohort identified at our institution. The TCGA cohort was primarily composed of male patients (70.5%) between the ages of 70 to 79 years (32.2%). Most patients were white (74.5%) and had stage 2 (37.1%) or stage 3 disease (36.8%). (Table S1 of Online Resource 1).

**Figure 1:**
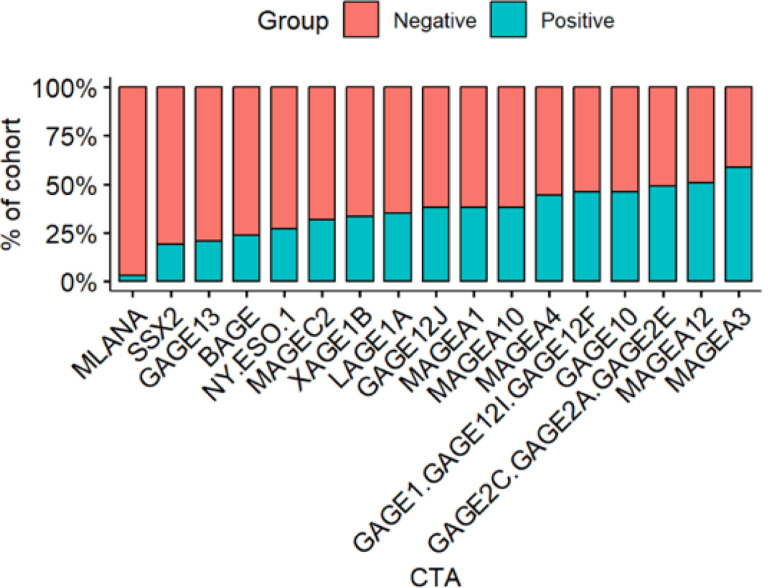
CTA prevalence in the Study Cohort Gene expression ranks of various cancer testis antigen genes in the study cohort.

The prevalence of CTA genes in the primary cohort is given in Fig. 1. The majority of genes range from a high of 58% for MAGEA3 to 19% for SSX2. MLANA, primarily a melanoma-associated gene, is an exception with a prevalence of only 3%. Apart from MAGEC2 (32%) and GAGE13 (21%), the MAGE and GAGE family of genes exhibit higher expression levels (38% - 58% and 38% - 49% respectively), while BAGE (24%), NY-ESO-1(27%), XAGE1B (33%) and LAGE1A (35%) show the opposite trend. In the TCGA cohort, MLANA was found the be the most expressed (95%) with GAGE12J being the least expressed (9%). Similar to the primary cohort, the MAGE family of genes was found to exhibit higher expression levels, ranging from 57% for MAGEA3 to 35% for MAGEC2. NY.ESO.1 (23%), BAGE (26%), and LAGE1A (28%) were again found to have lower expression levels. The prevalence of CTA genes in the TCGA cohort is given in Figure S1 of Online Resource 1.

**Figure 2:**
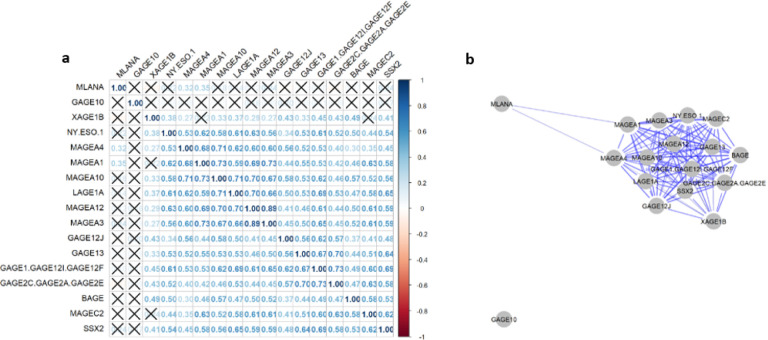
Study Cohort Co-expression Matrix and Network Plot **2a** Co-expression matrix of the CTA genes in the study cohort. Pairwise Pearson correlation r values are noted. **2b** Network plot of the CTA expression in the study cohort

**Figure 3:**
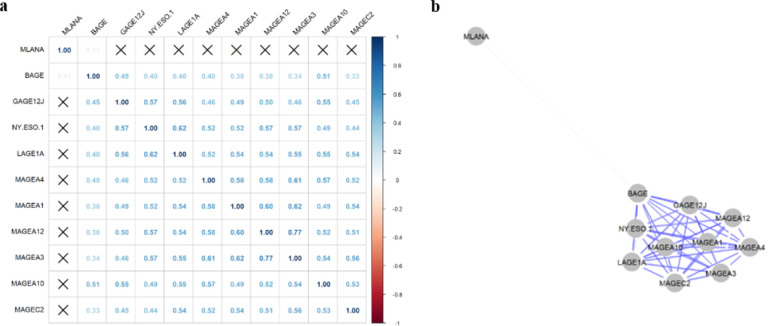
TCGA Cohort Co-expression Matrix and Network Plot **3a Co-**expression matrix of the CTA genes in the TCGA cohort. Pairwise Pearson correlation r values are noted. **3b** Network plot of the CTA expression in the TCGA cohort

We performed Pearson correlation among the seventeen CTA genes in the primary cohort and eleven genes in the TCGA cohort to identify co-expression clusters as potential targets of multimodal treatment. The correlation matrix as well as the network plot for the primary cohort are given in Fig. 2. We found a single cluster of genes as well as two genes separated from the others. The cluster includes MAGEA4, MAGEA3, NY.ESO.1, MAGEC2, MAGEA1, MAGEA10, MAGEA12, GAGE13, BAGE, LAGE1A, GAGE1.GAGE12L.GAGE12F, SSX2, GAGE12J, GAGE2C.GAGE2A.GAGE2E. XAGE1B is also part of the cluster but slightly offset. Both Gage10 and MLANA are separate from the cluster. The R^2^ Pearson correlation coefficient values in this cluster range from a low of 27 between XAGE1B and MAGEA4 to a high of 0.73 between the MAGE group of genes and the GAGE group of genes. Additionally, NY.ESO.1 had a high correlation with MAGEA1, MAGEA10, LAGE1A, MAGEA12, and GAGE1.GAGE12l.GAGE12F. In the primary cohort, MLANA was only correlated with MAGEA4, and MAGEA10 while GAGE10 was not co-expressed with any other CTA gene. The TCGA cohort showed similar results, displaying a single cluster of CTA genes with MLANA being separate (Fig. 3). The cluster in the TCGA cohort consisted of BAGE, GAGE12J, MAGEA12, NY.ESO.1, LAGE1A, MAGEA10, MAGEA1, MAGEA4, MAGEC2, AND MAGEA3. The R^2^ Pearson correlation coefficient values in this cluster range from a low of 0.33 between BAGE and MAGEC2 to a high of 0.77 between MAGEA3 and MAGEA2. Additionally, NY.ESO.1 showed high co-expression with MAGEA12, MAGEA3, LAGE1A, and GAGE12J. MLANA was only correlated with BAGE (R^2^ = 0.11).

We also performed survival analysis using the Kaplan-Meier method in the primary cohort as well as the TCGA cohort. In the primary cohort, we found that patients positive for MAGEC2 had significantly better survival than patients negative for MAGEC2 (positive vs. negative median survival in months, p-value; 31 months vs. 21 months, p = 0.034). We did not find any other significant trends with the other CTA genes. In the TCGA cohort, we found that patients with positive for MAGEA4 had significantly worse survival than patients negative for MAGEA4 (25 months vs. 32 months, p = 0.032). Additionally, patients positive for MAGEA1 had significantly worse survival than patients negative for MAGEA1 (23 months vs. 31 months, p = 0.013). One reason for the reversal of trends seen between the primary cohort and the TCGA cohort could be since the TCGA database was compiled prior to the introduction of immunotherapy. Therefore, we repeated the survival analysis in the primary cohort, splitting patients into subgroups depending on ICI treatment status.

Here, we found several significant results. In the non-ICI treated subgroup, MAGEC2-positive patients had worse overall survival than MAGEC2-negative patients (9 months vs. 30 months, p = 0.013). Similarly, GAGE2C.GAGE2A.GAGE2E-positive patients fared worse than GAGE2C.GAGE2A.GAGE2E negative patients (15 months vs. 29 months, p = 0.05). In the ICI-treated subgroup, GAGE13-positive patients tended to have better survival than GAGE13-negative patients, though the trend was not significant (72 months vs 13 months, p = 0.077). Similarly, SSX2 positive patients trended to better survival than SSX2 negative patients in the ICI-treated group (p = 0.068). MAGEA1 positive patients (34 months vs. 14.5 months, p < 0.01), MAGEA12 positive patients (34 months vs. 18.5 months, p = 0.043), MAGEA3 positive patients (34 months vs. 14.5 months, p = 0.028), MAGEA4 positive patients (72 months vs. 13 months, p < 0.01), and NY.ESO.1 positive patients (72 months vs. 18.5 months, p = 0.027) all had significantly higher overall survival than those negative for the corresponding CTA genes in the ICI-treated subgroup. The Kaplan Meier plots for the significant survival analyses are given in Figures S2, S3, and S4 of Online Resource 1.

## 4. Discussion

Gastroesophageal adenocarcinoma poses a significant threat, as existing systemic treatment options are limited in their efficacy. Therefore, it is essential to identify new therapeutic targets and develop treatments that specifically target these novel findings. One potential avenue of exploration involves targeting cancer/testis antigens (CTAs).

Before undertaking this task, a comprehensive understanding of the expression and co-expression landscape of cancer/testis antigens (CTAs) is crucial for identifying ideal targets for treatment. Unfortunately, data on CTA expression in gastroesophageal adenocarcinoma (GEAC) is currently limited. This study aims to fill this knowledge gap by investigating the expression patterns in a cohort of sixty-three GEAC patients through tumor sample gene expression analysis. Furthermore, the findings from our primary cohort were validated in a separate set of 329 patients extracted from The Cancer Genome Atlas. To our knowledge, this is the first study looking at CTA expression in GEAC. Some studies have explored CTA expression in cell lines, but none have explored this at a population level.

Our findings revealed that CTA genes exhibited expression levels ranging from 19–58% across patients. The patterns of expression were consistent across both the primary and TCGA cohorts, with the MAGE family of genes positioned at the higher end of the spectrum, while genes like BAGE, LAGE1A, and NY.ESO.1 demonstrated lower expression levels. An interesting anomaly was observed in the primary cohort, where MLANA was scarcely expressed, whereas in the TCGA cohort, it exhibited near-universal expression. This discrepancy might be attributed to MLANA primarily serving as a melanoma antigen, resulting in highly variable expression within esophageal and stomach tumors. Furthermore, our investigation revealed a significant level of co-expression among CTA genes. In both cohorts, CTA genes exhibited a cohesive cluster of expression, excluding MLANA. The MAGE family of genes exhibited the most consistent co-expression both within their own group and with other CTA genes, with Pearson R^2^ values ranging from 0.53 to 0.73. Likewise, similar patterns of co-expression were noted among the GAGE family of genes. Other genes like NY-ESO-1 and LAGE-1A exhibited more variable co-expression, with R^2^ values ranging from 0.37 to 0.71, which are still relatively high. These findings highlight that CTAs are fairly highly expressed in GEAC. Therefore, researchers can use this preliminary data to explore targeted interventions to address CTAs in GEAC. Moreover, despite the variability in CTA expression, many genes are significantly co-expressed. This suggests the feasibility of targeting multiple genes in individuals positive for a single gene, thereby enhancing the potential efficacy of therapeutic interventions. Notably, one of the most extensively studied CTAs in gastric and esophageal cancer includes the MAGE family of genes.

The Melanoma Antigen Gene family (MAGE) proteins were among the first identified members of cancer/testis antigens (CTAs) [26]. In 1995, Inoue et al. reported that at least one MAGE gene was expressed in 33 out of 42 esophageal tumor tissues, while none were expressed in 42 normal esophageal tissues [27]. Furthermore, Lian et al. found positive MAGE-A expression in 54.7% of resected gastric cancer patients, associating increased MAGE-A expression with poor differentiation, and a high clinical TNM stage [28]. In our analysis, we observed high expression and co-expression of MAGEA3, MAGEA12, MAGEA4, MAGEA10, and MAGEA1. Additionally, patients positive for MAGEA1, MAGEA12, MAGEA3, and MAGEA4 treated with immune checkpoint inhibitors (ICIs) demonstrated improved overall survival, suggesting that the MAGE family of genes presents an attractive target for developing targeted therapies. Targeted therapies against various MAGE antigens are still in phase I and II trials. A phase I trial investigated oncolytic virus therapy against MAGE-A3 in esophageal and gastric cancer patients, establishing safety and potential anti-tumor immune response [29]. This phase I trial established the safety of oncolytic virus therapy against MAGE-A3 as well as potential anti-tumor immune response. Another promising area of current research is T cell receptor therapy (TCR-T). In this, artificial expression of engineered T cell receptors (TCRs) in autologous T cells enables targeting of specific antigens [30]. Multiple trials targeting MAGE-A4 using TCR-T are in progress [11]. Preliminary results from a phase II trial (SURPASS) have shown an acceptable safety profile and evidence of antitumor activity in patients with gastroesophageal cancer after injection with next-generation ADP-A2M4CD8 SPEAR T-cells co-expressing the CD8a co-receptor with the engineered MAGE-A4c1032 T cell receptor (NCT04044859). Despite promising results from phase I and II trials, randomized controlled trials are required to translate these promising findings into clinical practice.

Shifting our focus to the use of CTAs as prognostic biomarkers, previous evidence has indicated that increased expression of CTAs is associated with worse overall survival in different cancer types[14, 31, 32]. In a cohort of 102 patients with intrahepatic cholangiocarcinoma, those with at least one CTA marker expression were significantly associated with worse overall survival[33]. Similarly, in esophageal squamous cell carcinoma, Sang et al. found that MAGE-A11 expression was an independent poor prognostic factor[34]. In gastric cancer, Lian et al. found that MAGE-A expression was associated with lymph node metastasis and poor 5-year overall survival but that it was not an independent prognostic factor [28]. In this study, we sought to understand the value of CTA expression as a prognostic biomarker. We did not see any consistently significant results in the two cohorts; however, we did see several trends when looking at individual CTAs. In the TCGA cohort, our analysis revealed that the overexpression of MAGE4 and MAGEA1 correlated with significantly reduced survival. Conversely, in our primary cohort, we observed that patients positive for MAGEC2 exhibited markedly better survival than those negative for MAGEC2. This incongruity may be attributed to the fact that 28% of the patients in the primary cohort received treatment with immune checkpoint inhibitors (ICIs), whereas no patients in the TCGA cohort underwent such treatment. We subsequently conducted a subgroup analysis of the primary cohort, stratifying patients based on ICI treatment status. In this analysis, we found that MAGEC2-positive patients not receiving ICI treatment experienced significantly reduced survival compared to MAGEC2-negative patients, similar to the TCGA cohort. Interestingly, among patients treated with ICIs, those positive for GAGE13, SSX2, MAGEA1, MAGEA12, MAGEA3, MAGEA4, and NY.ESO.1 all demonstrated significantly higher overall survival than those negative for the corresponding CTA genes. This aligns with the notion that CTA overexpression is associated with worse survival but also suggests that they present attractive targets for immunotherapy, potentially leading to increased treatment response. In a recent study, Seager et al. demonstrated a significantly improved overall survival rate in pembrolizumab-treated lung cancer patients with increased CTA burden[35].

A significant caveat to our results is that this is a single institution retrospective study and further prospective studies are required to evaluate this association. Other limitations of our study include small patient numbers, and lack of information on disease stage (treated or untreated), stage at diagnosis, and biopsy site.

## 5. Conclusion

To the best of our knowledge, this study represents the first comprehensive characterization of cancer/testis antigen (CTA) expression and co-expression in a clinical cohort of gastroesophageal adenocarcinoma (GEAC) patients. Apart from identifying a relatively high prevalence of CTA expression, our analysis unveiled notable co-expression among CTA genes. This opens avenues for researchers to explore specific targeted interventions based on this data. However, whether CTAs can serve as prognostic biomarkers for GEAC remains uncertain, and further research, including prospective trials, is necessary to investigate this aspect. These findings are hypothesis-generating, and we propose that future preclinical and clinical investigations should delve into the possibility of targeting multiple CTA genes, as well as examining the potential of CTAs as biomarkers in gastroesophageal adenocarcinoma.

## Figures and Tables

**Table 1:** Demographic and Disease Characteristics of the Study Cohort

Characteristics	n (%) (N = 63)
**Age at Initial Diagnosis**	
30–39	5 (7.9%)
40–49	4 (6.3%)
50–59	12 (19%)
60–69	24 (38.1%)
70–79	16 (25.4%)
≥ 80	2 (3.2%)
**Gender**	
Male	55 (87.3%)
Female	8 (12.7%)
**Race**	
White	59 (93.7%)
Black	2 (3.2%)
Asian	1 (1.6%)
American Indian	1 (1.6%)
**BMI**	
< 25	18 (28.6%)
≥ 25	45 (71.4%)
**Stage**	
1	2 (3.1%)
2	6 (9.5%)
3	8 (12.7%)
4	40 (63.5%)
No Data	7 (11.1%)
**Tumor Location**	
Stomach	15 (23.8%)
Esophagus	37 (58.7%)
Gastroesophageal Junction	11 (17.5%)
**Primary or Metastatic**	
Primary	41 (65.1%)
Metastatic	21 (33.3%)
No Data	1 (1.6%)
**PD-L1 IHC Status (CPS ≥ 1)**	
High	39 (61.9%)
Low	14 (22.2%)
No Data	10 (15.9%)
**Immunotherapy Received**	
No	45 (71.4%)
Yes	18 (28.6%)

Demographics of the patient population in the study cohort including age, gender, race, and BMI are listed above. Characteristics of the disease including tumor location, stage of cancer, primary/metastatic, PD-L1 IHC status, and ICI treatment status are also listed. All values are given as total numbers and percentages.

* N = number, BMI = body mass index, PD-L1 = Programmed death ligand-1, IHC = Immunohistochemistry, CPS = Combined positive score

## Data Availability

The de-identified data is available with the research team and can be made available on request by contacting the corresponding author at sarbajit.mukherjee@roswellpark.org.

## Key Points

Cancer/testis antigens are highly expressed in GEACs with MAGEA3 being the most frequently encountered gene. There is a significant correlation between the expression of MAGE A3 and NY-ESO-1. We propose targeting multiple CTAs for effective tackling of advanced malignancies.
